# The potential of using biotechnology to improve cassava: a review

**DOI:** 10.1007/s11627-016-9776-3

**Published:** 2016-08-12

**Authors:** Paul Chavarriaga-Aguirre, Alejandro Brand, Adriana Medina, Mónica Prías, Roosevelt Escobar, Juan Martinez, Paula Díaz, Camilo López, Willy M Roca, Joe Tohme

**Affiliations:** 1 Agrobiodiversity Research Area, International Center for tropical Agriculture-CIAT, AA 6713 Cali, Colombia; 2 Biology Department, Universidad Nacional de Colombia, Carrera 30 No. 45-03. Edificio 421, Bogotá, Colombia; 3 International Potato Center-CIP, Av. La Molina 1895, Lima 12, P.O. Box 1558, Lima, Perú

**Keywords:** Transgenic cassava, *Ensifer*-mediated transformation, Somatic embryogenesis, Cassava viruses whiteflies CBB, Effector binding element EBE, Artificial TALs, Nutritional improvement, Propagation, Synthetic seeds, Genome editing, Climate change

## Abstract

The importance of cassava as the fourth largest source of calories in the world requires that contributions of biotechnology to improving this crop, advances and current challenges, be periodically reviewed. Plant biotechnology offers a wide range of opportunities that can help cassava become a better crop for a constantly changing world. We therefore review the state of knowledge on the current use of biotechnology applied to cassava cultivars and its implications for breeding the crop into the future. The history of the development of the first transgenic cassava plant serves as the basis to explore molecular aspects of somatic embryogenesis and friable embryogenic callus production. We analyze complex plant-pathogen interactions to profit from such knowledge to help cassava fight bacterial diseases and look at candidate genes possibly involved in resistance to viruses and whiteflies—the two most important traits of cassava. The review also covers the analyses of main achievements in transgenic-mediated nutritional improvement and mass production of healthy plants by tissue culture and synthetic seeds. Finally, the perspectives of using genome editing and the challenges associated to climate change for further improving the crop are discussed. During the last 30 yr, great advances have been made in cassava using biotechnology, but they need to scale out of the proof of concept to the fields of cassava growers.

## Introduction

Cassava (*Manihot esculenta* subspecies *esculenta Crantz*; Euphorbiaceae) is native to the South American tropics were its closest wild relative *M. esculenta* ssp. *flabellifolia* (Pohl) has been reported and studied to determine its degree of relationship with cultivated cassava (Olsen and Schaal 1999; Léotard et al. 2009). Since its introduction in West Africa by Portuguese sailors in the sixteenth century, cassava expanded throughout the tropics, especially in sub-Saharan Africa, India, the Philippines, and Indonesia, where today it represents a source of food and income for over 800 million people worldwide. The importance of cassava as a food and industrial crop relies on its roots since they accumulate starch (approximately 30–60% dry matter), and so, it is considered the second source of starch globally, after maize (FAO 2013). Cassava can be grown in marginal soils, typical of low-income, small-scale farmers, with minimum input and without the need of predictable rainfall.

The average yield of cassava worldwide is only 12–13 tons/ha, but its potential yield under optimal conditions is almost seven times larger (80 tons/ha; FAO 2013). According to FAO statistics (FAOSTAT 2015), cassava world production raised to >263 million tons in 2013, a 27% increase in production during the last 10 yr. From these, Asia contributed 33.5% (88.2 million tons), Africa 54.8% (144.2 million), and the Americas 11.6% (30.5 million tons). Among these three regions, Asia holds the highest average yield per hectare at 21.1 tons, still far from its true yield potential, followed by the Americas (12.3 tons) and Africa (8.3 tons). Thus, this trend indicates that Asia will continue growing in production and yield, while Africa, constrained mostly by viral diseases that affect the crop severely, is likely to increase the area of planting in the coming years. Meanwhile, production and yield in the Americas seems to be going downwards, mainly as a result of uncompetitive production costs. Besides having cheaper human labor costs, Asia set up the growing production trend by adopting high-yielding, high-starch cassava varieties, and better agronomic practices for fertilization and soil protection that resulted in very competitive production costs and higher per-capita consumption in the region. With the European and Chinese markets secured, and the world population growing at the actual pace—Africa’s population is expected to double to 2.4 billion for 2050, the prospects for cassava’s increase in production are therefore very promising in Asia and Africa. In spite of yield constrains in Africa, Nigeria is still the major cassava world producer with 47.4 million tons in 2013, followed by Thailand (30.2) and Indonesia (23.9). The roots of cassava can be harvested as early as 8 mo, and rarely as late as 14–16 mo after planting, although ideally roots are harvested within the 12 mo following seeding. Thailand, Vietnam and India have specialized in the production and processing of cassava for animal feed, with the European countries being the main consumers, followed closely by China that today is the main importer of dried cassava and starch (FAO 2013). China and Thailand, on the other hand, are realizing the potential of cassava for the production of bioethanol, a relatively new role for the crop that holds great promise (Nguyen et al. 2007; Jansson et al. 2009; Cortés-Sierra et al. 2010).

Clonal propagation of cassava facilitates the free exchanging of stems between farmers for planting, yet it also facilitates the spread of diseases, especially bacteria, fungi, phytoplasmas (mycoplasma-like organisms), and insects hosting harmful viruses that cause two of the most cassava’s devastating diseases: cassava mosaic disease (CMD) and cassava brown streak disease (CBSD; reviewed in Legg et al. 2014). The first efforts to incorporate biotechnology as a tool for improving cassava took place some 30 yr ago, possibly with the discovery of regeneration through somatic embryogenesis and clonal propagation (Stamp and Henshaw 1982; Szabádos et al. 1987). But, the truth is that cassava farmers are still lacking genetically modified (GM) varieties to help them overcome the many hurdles for its cultivation. Those GM varieties already exist, below, but are still at the proof-of-concept stage, in the field-testing phase, possibly to make sure that transgenic traits persist in time. The purpose of these comments is to encourage optimism among farmers, consumers, and/or researchers regarding their expectations for biotechnological solutions for cassava. Many optimistic cassava scientists still pursue GM varieties and, as it will be described below, there are encouraging cases in which significant progress has been made. As examples, biotechnology applied to the control of virus diseases, *in vitro* propagation, synthetic seed production, and the enhancement of the roots´ nutritional value will be discussed. These cases all have the potential to provide a biotechnological solution for the improvement of cassava. This approach has been recognized and reviewed in the recent past (i.e., Fregene and Puonti-Kaerlas 2002; Taylor et al. 2004; Liu et al. 2011).

### History of the first transgenic cassava plant

Genetic transformation of cassava (*M. esculenta* Crantz) using *Agrobacterium tumefaciens* or particle bombardment as gene-delivery systems is a reality after more than 25 yr of continuous efforts of several labs worldwide. With both systems, it has been possible to obtain transgenic plants of cassava expressing marker and selectable genes, as well as genes of agronomic interest. However, *Agrobacterium*-mediated transformation (Agrotrans) of cassava has been the technology of choice because it is more easily accessed by national agricultural research programs (NARPs) in developing countries, where ultimately, transgenic cassava landraces with novel traits are most needed. Agrotrans produces fewer and cleaner insertions of transfer DNAs (T-DNAs), which facilitates the safe release and commercialization of transgenic plants. The reader is encouraged to check the following publications if interested in transformation of cassava using micro-particle bombardment: Schöpke et al. (1996); Raemakers et al. (1996); Zhang et al. (2000), and Zhang and Puonti-Kaerlas (2000).

Although the first genetic transformations of cassava using *Agrobacterium* were published in 1996 (Li et al. 1996; Raemakers et al. 1996; Schöpke et al. 1996), much work was done prior to these reports, especially at the International Center for Tropical Agriculture (CIAT) and at the Vrije Universiteit Brussel. The pioneering experiments that culminated with the production of the first transgenic cassava calli, expressing selectable and useful genes, were developed towards the end of the 1980s by (Calderon-Urrea 1988). In these experiments, Calderón-Urrea transformed somatic embryos from the cultivar Mcol1505 with several *A. tumefaciens* strains to introduce resistance to the herbicide phosphinothricin (PPT; commercially known as Basta^®^ or Finale^®^) using the *bar* gene. Additionally, the *uid*A gene for β-glucuronidase expression, also known as GUS gene, was introduced. The presence of these genes in calli was demonstrated by Southern blot analysis. Thus, this was the first demonstration, at the phenotypic and molecular level, of the expression of foreign bacterial genes in cassava cells. At the beginning of the 90´s another series of transformation experiments were initiated using a wild type *Agrobacterium* strain, named CIAT-1182, isolated from cassava plants grown in the field (Sarria et al. 1993), to introduce the same *bar* gene (plasmid pGV1040 from PGS) into somatic-embryo-derived cotyledons (SEDCs; Arias-Garzón and Sarria 1995) of the cultivar MPer183. Several putatively transgenic lines (based on GUS expression and PCR tests and PPT selection) were rescued, although only in one of them (line 53-5.2), the insertion of the T-DNA in at least three different sites was proved at the molecular level. Basta^®^ spraying of plants in the greenhouse showed that line 53-5.2 was highly resistant to the herbicide. Thus, the first transgenic plants of cassava were so established, expressing a gene of potential commercial use. Roca’s transformation team at CIAT first announced their results at the second meeting of the Cassava Biotechnology Network (CBN), held in Indonesia in August of 1994 (Sarria et al. 1995), and then published them in a peer-reviewed journal in 2000 (Sarria et al. 2000).

**Figure 1.**
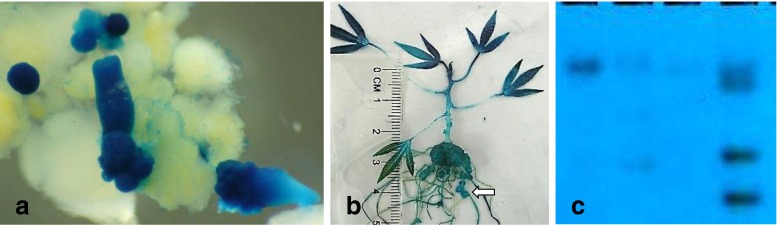
(*a*) Transgenic somatic embryos and (*b*) plant of cassava cv. 60444 transformed with *Ensifer adaherens* OV14, expressing GUS. Note the formation of nodules on roots (*arrows*). This event was one of the three obtained for which a Southern blot (*c*), confirmed the presence of single copy insertions (*first and third lanes*) as well as multicopies of the T-DNA (*second lane*; *fourth lane* is control transgenic plant).

More recently, cassava has been transformed with bacteria different than *Agrobacterium* named *Ensifer adhaerens* OV14. It contains chromosomal genes homologous to virulence genes of *Agrobacterium* (Rudder et al. 2014) and was identified in 1982 as a gram-negative, predatory bacterium, inhabiting the rhizosphere with the ability to transfer genes into several plants, i.e., potato, tobacco, *Arabidopsis*, *Solanum*, and rice (Casida 1982; Wendt et al. 2012; Soto et al. 2015). Apparently, *Ensifer* seemed to be less virulent and pathogenic than *Agrobacterium* and therefore was considered an ideal vector to produce clean and unique insertions into plants (Rudder et al. 2014; Zúñiga-Soto et al. 2015). The Genetic Transformation Platform at CIAT used *E. adhaerens* strain OV14 with plasmid pCAMBIA5105 to transform cassava cv. 60444, based on the protocol reported by Zúñiga-Soto et al. (2015) for rice. Three transgenic independent lines were confirmed by Southern blot as having one insert (two lines) or four copies of the T-DNA (Fig. 1). Expression of the GUS gene was evident in the single-copy events. In all transgenic plants, the appearance of nodules in roots was clearly visible (Fig. 1), an observation previously made by Rogel et al. (2001). Thus, cassava entered the list of crops that can also be transformed with *Ensifer adaherens*.

### Molecular aspects of somatic embryogenesis and friable embryogenic callus induction: implications in genetic transformation and genome editing

Since the first reports on cassava transformation during the early 1990s (i.e., Sarria et al. 1993; 1995; Schöpke et al. 1993), the development of transgenic technologies has emerged as a promising strategy for improving the crop by overcoming limitations related to conventional breeding and accelerating the incorporation of agronomic characteristics (Fregene and Puonti-Kaerlas 2002; Taylor et al. 2002). However, several constraints still remain, which prevent applying these biotechnological approaches to farmer and industry-preferred landraces. One of the principal limitations is related to the production of friable embryogenic callus (FEC), the target tissue most efficient and widely used in cassava genetic modification to date (Taylor et al. 1996; Bull et al. 2009; Liu et al. 2011). Producing FEC is the result of an arduous process that involves about 4 to 6 mo, depending upon genotype and tissue culturist’s experience. It includes a series of steps including: propagation of *in vitro* plant material, primary somatic embryogenesis (SE) induction, secondary cyclic SE and FEC induction, isolation and purification through a continuous subculturing process (Bull et al. 2009; Taylor et al. 2012). Embryogenic material is induced by culturing embryogenic-competent tissue such as immature leaf lobes or axillary buds. The culture medium is commonly supplemented with auxins, mainly picloram. FEC is induced by transferring the organized embryogenic structures (OES) onto Gresshoff and Doy (Gresshoff and Doy 1974) DBM2 medium supplemented with 50 μM picloram (Taylor et al. 1996). Even though all this process has been well established for the model benchmark genotype 60444, their applicability to other cultivars has been limited because FEC generation is strongly genotype-dependent, meaning that embryogenic efficiency varies significantly between genotypes (Liu et al. 2011). During the last 6 yr the development of more robust and efficient transformation protocols for 60444 (Bull et al. 2009; Taylor et al. 2012) has represented the starting point to incorporate more cassava landraces into pipelines for genetic modification (Table 1). This has required modifying protocols to overcome constraints like limited amount and low-quality FEC, wide variation in recovery and proliferation time on selective media, and the extremely time-consuming purification of new FEC from friable, non-embryogenic callus (Zainuddin et al. 2012; Chetty et al. 2013; Nyaboga et al. 2013).

**Table 1.** Transgenic cassava cultivars reported since 2010 for which genes expressing traits of interest for producers and/or consumers, other than marker and selectable genes, have been introduced

Source	Cassava genotypes	Traits of interest (genes)
Welsch et al. (2010)	60444	Biofortified β-carotene (*crt*B)
Bonilla (2010)	60444	Biofortified β-carotene (*crt*B, *crt*I, *crt*Y)
Zhang et al. (2010)	60444	Leaf retention (senescence-inducible *ipt*)
Zhao et al. (2011)	60444	Waxy starch (RNAi GBSSI)
Yadav et al. (2011)	60444	CBSVD (RNAi FL-CP)
Narayanan et al. (2011)	60444	Protein content/cyanogenic content (HNL)
Taylor et al. (2012)	60444	RNAi CMD (ACMV/EACMV); CBSD (n.d.)
Ihemere et al. (2012)	60444^z^	Iron biofortification (*FEA1*)
Vanderschuren et al. (2012)	TME7 (Oko-Iyawo)	CMV and CBSV resistance (RNAi-CBSV coat protein)
Koehorst-van Putten et al. (2012)	Adira4	Waxy starch (RNAi-GBSSI)
Ogwok et al. (2012)	60444	UCBSV resistance (siRNA-UCBSV coat protein)
Failla et al. (2012)	60444	Biofortified β-carotene (*crt*B and DXS)
Odipio et al. (2014)	60444	UCBSV resistance (RNAi-UCBSV coat protein)
Ntui et al. (2015)	KU50^z^	Resistance to Sri Lankan CMV (AV2 and AV1 coat proteins)
Narayanan et al. (2015)	TME 204	Iron biofortification (*At*VIT1)
Chauhan et al. (2015)	TME 204, TME7, 60444	Resistance to CBSV and UCBSV, increase carotene content in roots
Li et al. (2015)	60444	Biofortified vitamin B6 (*At*TDX1.1 and *At*TDX2)
CIAT 2015 (this review)^y^	60444, SM1219-9	Centromere-engineering for haploid induction and herbicide (PPT) tolerance (RNAi-CENH3 and modified versions of CENH3 plus *bar*)^x^; tolerance to PPD (root-specific SOD)^w^; resistance to *Xanthomonas* (RNAi-RXam1)^v^; stomatal opening (guard cell-specific AtAHA2); flowering induction (phloem-specific Hd3a from rice)

Other reports on transgenic cassavas previous to 2010 were reviewed by Liu et al. (2011)

^z^Cultivars transformed using SEDCs rather than FEC

^y^Over 260 Southern-positive, transgenic lines have been produced, some currently on field trials or in the greenhouse

^x^In collaboration with A. Britt, UC-Davis, USA

^w^In collaboration with P. Beyer, Univ. of Freiburg, Germany

^v^In collaboration with C. López, Univ. Nacional de Colombia

More recently, researchers have managed to produce genetic transformation using FEC in cultivars different than 60444, including African farmer- and industry-preferred landraces such as TME3, TME7, TME14, TME204, T200, Ebwanatereka, Kibandameno, and Serere (Vanderschuren et al. 2012; Zainuddin et al. 2012; Chetty et al. 2013; Nyaboga et al. 2013, 2015; Chauhan et al. 2015). This is encouraging but still represents only a small fraction of the more than 5000 varieties recognized for cassava (Salvador et al. 2014). Nonetheless, not only obtaining FEC represents a hurdle, also FEC’s dividing capacity is critical to produce large amounts of tissue to ensure the production of enough transgenic lines (Hankoua et al. 2006; Zainuddin et al. 2012). Additionally, the subculturing process for FEC proliferation generates high levels of somaclonal variation due to extended exposure to auxin. This in turn produces changes in the endogenous hormone metabolism (Raemakers et al. 2001; Taylor et al. 2001; Bull et al. 2009), which constitutes a problem because it impacts negatively the plant conversion ability of embryos and the morphology of regenerated plants. Thus, labs are forced into the staggered and continuous production of fresh FEC lines almost monthly, which is expensive time and manpower wise.

The production of FEC for the elite Asian line Kasetsart University 50 (KU50), the most widely grown in Asia for biofuel production (Ntui et al. 2015), has not been well documented to our knowledge. Ma et al. (2015) stated that it was actually produced with low efficiency, probably meaning that induction of the primordial FEC was possible but its proliferation may not be possible. Researchers at CIAT have made similar observations by developing KU50’s FEC primordia on media containing tyrosine, but cell division slowed after the first FEC purification steps (unpublished). This may be one of the reasons why KU50 has been transformed using SEDCs instead of FEC (Ntui et al. 2015).

Until now, the approaches used to produce FEC in new cassava varieties has focused on identifying the correct media composition. For example, including amino acids like l-tyrosine in the culture medium resulted in the production of FEC for several African cultivars (Nyaboga et al. 2013; Chauhan et al. 2015). The use of DKW/Juglan’s salts (Driver and Kuniyuki 1984) enhanced somatic embryogenesis in the cultivars TME14 and TME204 (Chauhan et al. 2015; Nyaboga et al. 2015). Decreasing auxin concentration improved somatic embryo differentiation in the African variety Ebwanatereka (Apio et al. 2015). On the other hand, the efficiency of transformation of FEC has also been improved by modifying the optical density of *Agrobacterium* or by adding cephalosporins prior to inoculation with bacteria (Chauhan et al. 2015). Thus, modifications in either tissue culture components, physical treatments like hovering OES (Taylor et al. 2012) and changes in *Agrobacterium* density have been useful to expand the production and transformation of FEC to more genotypes.

Nevertheless, due to the high heterozygosity of cassava, and given the high variation in FEC production, the development of standard SE protocols for each genotype constitutes a laborious and time-consuming task that requires well-trained tissue culturists, large amounts of plant material, media, chemicals, and infrastructure (Bull et al. 2011; Zainuddin et al. 2012). All the above requirements may generate yield gaps for technology transfer, e.g., to laboratories in developing countries where cassava is a staple food and source of income.

The molecular mechanisms behind FEC production are just being elucidated. The recent publication by Ma et al. (2015) made a robust analysis at histologically, metabolic, epigenetic, and expression-profiling levels of FEC formation to identify the molecular regulatory networks involved. This research found a wide set of differentially expressed genes in FEC samples related to SE. It also linked the decrease in DNA methylation, the upregulation of cell cycle-related genes, and the change in expression of certain transcription factors to the high somaclonal variation observed in long-termed, subcultured FEC.

The use of embryogenesis marker genes (EMGs) for inducing SE to transform cassava has not been explored yet. Controlling the expression of EMGs may be an alternative to regenerate SE after transforming tissues, thus overcoming the limitation to produce FEC. In different plant species, it has been shown that overexpression of certain EMGs can lead to the formation of somatic embryos in vegetative cells (Ikeuchi et al. 2013). However, little is known about the molecular mechanisms of somatic or zygotic embryogenesis in cassava. No studies have been published describing key EMGs. In general, it is well known that *in vitro* SE in plants is affected by a large set of conditions including genotypes, explant types, general *in vitro* settings, and plant growth regulators (PGR), among other factors (Zimmerman 1993; Mordhorst et al. 1997). Altogether, they create the inducing environment that triggers the embryogenic process leading to the dedifferentiation of somatic cells, followed or paralleled by the reacquisition of developmental totipotency (reviewed by Feher 2015). This process is highly regulated by genetic and epigenetic mechanisms, the latter being responsible for changing the overall expression of genes, integrating stress with hormonal and developmental pathways (Ikeuchi et al. 2013; Feher 2015).

As a first approach to understand SE in cassava, Baba et al. (2008) used histology to show that somatic embryos can be produced from procambium vascular tissue, which constitutes a region hosting competent embryogenic cells with high mitotic activity. They also identified a set of proteins expressed in somatic tissues undergoing secondary SE that were involved in a wide range of metabolic functions. However, up to date, there is no report of a molecular approach seeking embryogenesis-specific regulatory genes in cassava. Recently, CIAT started working on the identification and characterization of *Arabidopsis* LEAFY COTYLEDON 1 (LEC1) and LEC2 orthologous genes in the genotype 60444. These transcription factors (TFs) are considered master regulators of embryogenesis due to their multifunctional role in SE. They induce other transcription factors and proteins that control developmental and metabolic pathways (reviewed by Braybrook and Harada 2008). CIAT’s preliminary results indicate that LEC TFs candidate genes have a highly conserved molecular function in cassava and are probably involved in the transition from a somatic to an embryonic state (Brand et al. 2016).

Efforts to elucidate EMGs are not unique to cassava. It is also the approach for cocoa. BABY BOOM (TcBBM), a transcription factor, is being used to enhance SE following transient expression promoting clonal propagation of elite varieties while improving regeneration of cocoa transgenic plants (Florez et al. 2015). However, what is known from *Arabidopsis* is that SE is a complex, well-coordinated process wherein several factors are involved; therefore, it is likely that more than one EMG will be required for manipulating SE.

Somatic embryogenesis, the underlying mechanism of FEC induction, has been one of the most successful biotechnological tools used for genetic modification and large-scale propagation of crops. In the case of cassava, FEC has led the way to address questions about crop biology and breeding through proof of concept experiments. Using FEC has the advantage of preserving the heterozygosity of cassava, maintaining desired characteristics, with exceptions (see the following section). For most traits evaluated in the field, SE does not seem to generate undesirable variability.

From a biotechnological perspective, a deep understanding of SE and FEC production represents a challenge to improve methods for cassava transformation, but certainly it will not be the only way for breeding cassava using biotechnology. Currently, the new genome editing tools, like the CRISPR-Cas9 system (Osakabe and Osakabe 2014), represents a milestone not only for highly specific and efficient genome edition but also for the possibility of generating modified/edited plants which bypass the overwhelming regulation for transgenics (Waltz 2016). The new perspective focuses on obtaining products without foreign DNA such as selection marker genes, e.g., for resistance to antibiotics and/or herbicides, or sequences from viruses and bacteria. Thus, new approaches in genetic modification and gene editing introduce preassembled CRISPR-Cas9-sgRNA, a ribonucleoprotein, into plant protoplasts to edit genes (Woo et al. 2015), avoiding using *Agrobacterium* as vector to deliver DNA. Even if foreign DNA was introduced using vectors, the cassette containing Cas9 and guide-RNAs can be segregated out of the edited plants in the following generation. This is commonly the case for rice, but not for cassava where sexual reproduction changes the genotype due to recombination. Then, protoplasts should be used if editing is the focus. A protocol for isolation and regeneration of protoplast has been already described since the 1990s (Sofiari et al. 1998), which opens the way for testing genome editing in cassava using the CRISPR-Cas9 system.

### Resistance to viruses and whiteflies

In the case of transgenic resistance to viral diseases, this review does not ignore the tremendous amount of research done to understand how RNAi-mediated resistance would work in cassava, particularly approaches involving posttranscriptional silencing of Coat Protein (*CP*) genes, or the AC1 (*Rep*), AC2 (*TrAP*), or AC3 (*REn*) genes implicated in replication of viral DNAs, in the model cassava genotype 60444 (reviewed by Liu et al. 2011). Some of these studies are referenced in Table 1, for example, Vanderschuren et al. (2012), among others. However, the following text emphasizes cases where the same or similar strategies have been applied to produce transgenic landraces or elite lines that, for obvious reasons, have tremendous value for cassava farmers, especially in Africa and Asia. It is worth clarifying that some authors consider the genotype 60444 a West African cultivar (Taylor et al. 2012); therefore, it was included in Table 1.

Like most transgenic crops, the genetic transformation of cassava has been limited by the difficulty in efficient production of transgenic landraces expressing genes of interest. In terms of landrace-specific protocol development to produce transgenic lines for field-testing, the progress seems moderate though important. This indicates that researchers have been able to reduce the recalcitrance of the crop to transformation and regeneration, to the point that transgenic lines of farmer-preferred cultivars have even reached field trials. As an example, the transgenic cassava landrace Adira4, with waxy starch, was the first tested in Indonesian fields, probably several years before it was actually published (Koehorst-van Putten et al. 2012). The transgenic cassava genotype that held promise was a Nigerian landrace, naturally resistant to CMD called TME7, for which engineered resistance to cassava brown streak virus (CBSV) and Ugandan-cassava brown streak virus (UCBSV) was incorporated (Vanderschuren et al. 2012). More recently, Chauhan et al. (2015), also introduced resistance to UCBSV, the causal agent of CBSD, and enhanced the nutritional quality of roots of the African landraces TME204 and Oko-Iyawo (TME7). Similarly for Asian varieties, Kasetsart University 50 (KU50) is an elite line widely grown by many farmers in this continent for its high dry matter content. However, it is highly susceptible to CMD caused by the Sri Lankan cassava mosaic virus (SLCMV; Dutt et al. 2005). The good news is that resistance to SLCMV has been engineered in KU50 by RNAi-mediated silencing of the AV1 coat protein and AV2 pre-coat protein genes, resulting in at least four single copy events that turned out to be highly resistant to SLCMV (Ntui et al. 2015).

No doubt that all aforementioned new transgenic landraces could be candidates to move out of confined field trials into multi-site, open-field testing because, as stated by Legg and collaborators in their 2014 review on virus diseases of cassava, the stability of engineered resistance to CMD or CBSD has to be demonstrated in the field, over several cycles of clonal propagation (Legg et al. 2014). However, recently it was reported that the production of transgenic and non-transgenic cassava plants through SE and/or FEC resulted in loss of the most important trait for Africa and Asia: resistance to CMD. Indeed, African cultivars, i.e., TME204, TME3, and TME7, carrying a monogenic, dominant, non-transgenic resistance called CMD2 (Legg et al. 2014), lost resistance to CMD after being regenerated *in vitro* via somatic embryogenesis (Beyene et al. 2015). The cause of such loss is unknown, but predictably to be of epigenetic nature. The news highlighted the importance of doing deep phenotypic analysis for the most promising transgenic lines, for any trait, analyses that must score morphological and agronomic descriptors (Fukuda et al. 2010) in search of phenotypic variants as indicators of probable epigenetic variation. Epigenetic analyses may also help in the early detection of tissue culture variants, for example, to estimate the magnitude of changes in the methylome, which varies among conventional and tissue-culture propagated plants (Kitimu et al. 2015). The implication of the finding is clear for those improving traits through biotechnology in African and Asian cultivars with CMD2-type resistance: they must find alternative methods to regenerate transgenic plants that avoid somatic embryogenesis or FEC or start working with cultivars with CMD1- and/or CMD3-type of resistances. Indeed the search for such new methods is already started. Researchers at the Donald Danforth Plant Science Center in St. Louis, MO, used the cytokinin meta-topolin [6-(3-hydroxybenzylamino) purine] to induce *in vitro* shoots on non-embryogenic explants of several cassava cultivars of African, American, and Asian origin (Chauhan and Taylor 2016).

Nevertheless, the status of functional biosafety regulatory bodies in African countries is unclear and therefore the successful completion of these stories may be at stake. With the exception of South Africa, Burkina Faso, and Sudan where GM crops were grown in 2014 (James 2014), approval of their commercialization, meaning trade, production, importation, planting, processing, etc., is still pending. Even in Uganda and Kenya where GM bananas, cotton, cassava, and drought- and insect-tolerant maize have been tested in confined field trials (James 2014; Nang’ayo et al. 2014), GM crops have not been released yet. We can only hope that decision makers in these countries impose the needs of cassava farmers over the interests of anti-GM advocates.

The success of transgenic technology with major crops such as corn, soybeans, canola, and cotton, for which transgenic plants have temporarily solved the problem of susceptibility to lepidopteran pests, is undeniable (James 2014). For cassava, this solution is still to come, but hope remains, especially to fight against whiteflies, which are without doubt the most damaging insect pests of cassava in the three continents where it is grown. White fly attack causes hunger, famine, and losses that surpass a billion of dollars per year. Whiteflies are hemipteran insects that feed from the phloem by breaching plant tissues to retrieve nutrients. *Bemisia tabaci* in Africa transmits the causal viral agent of CMD, and *Aleurotrachelus socialis* in South America and the Caribbean causes damage by direct feeding. *A. socialis* has recently being implicated also in the transmission of a still unidentified agent causing cassava frog skin disease (CFSD) in Colombia (Carvajal-Yepes et al. 2014; Legg et al. 2014), for which there seems to be a phytoplasma implicated as well (Alvarez et al. 2009). A biotech approach likely to combat insect pests of cassava must focus on biological systems for which there is enough molecular information, such as molecular mechanisms of plant defense, the metabolic pathways and genes involved. Whiteflies are beginning to fit these requisites. The laboratory of Linda Walling in Riverside (CA) found that the effective development of nymphs of *B. tabaci* type B on *Arabidopsis thaliana* relies on the activation of the salicylic acid-defense pathway (SA pathway) and, simultaneously, the decline or unchanged RNA levels of genes involved in the jasmonic acid/ethylene (JA/ET) defense signaling mechanism (Zarate et al. 2007; Walling 2008). On the other hand, findings by Bohórquez’s laboratory at CIAT, working with nymphs and adults of *A. socialis* feeding on the whitefly-resistant cassava landrace Ecu72, identified genes that were either upregulated (310) or down-regulated (210). Among the induced genes were chitinases, lipoxygenases, and methyl-transferases like cafeoyl-CoA-o-methyltransferase, the latter being a gene involved in lignin synthesis. Reinforcing cell strength by extra deposition of lignin on the wall during insect attacks may preclude sucking insects from probing cells with their stylets thus avoiding virus transmission. Similarly, the mRNA of LOX5 accumulated in whitefly-infested Ecu72 (Bohórquez 2011). Given that salicylic acid, jasmonic acid, and ethylene control several of the cellular biochemical paths that respond to pathogens and pests, proof that individual genes from both pathways confer resistance to whiteflies in cassava, seems crucial. The transgenic-mediated overexpression and/or down-regulation of genes like LOX5 or cafeoyl-CoA-o-methyltransferase in cassava, aiming for the development of new cassava varieties resistant to whiteflies, is a logical biotechnological approach to demonstrate the role of these genes in cassava’s defense against whiteflies. In fact, collaborative research between research institutes, universities and organizations of 11 countries, under the umbrella of the African Cassava Whitefly Project (2015), funded by the Bill and Melinda Gates Foundation, is underway to use genomics, proteomics and metabolomics to better understand whitefly systematics and its outbreaks, cassava resistance to both whiteflies and viruses, and to generate social and economic data for impact assessment.

### Exploiting plant-pathogen Interaction as a tool to improve cassava traits

One of the major constraints in cassava is yield losses caused by diverse viral and bacterial diseases. *Xanthomonas axonopodis* pv. *manihotis* (*Xam*) is the causal agent of cassava bacterial blight (CBB), the main devastating bacterial disease in cassava. Depending on environmental conditions, CBB can cause field losses of up to 75% (Lozano 1986; Wydra and Verdier 2002). *Xam* is so important that, according to the Molecular Plant Pathology Journal, today it is considered one of the most relevant plant pathogenic bacteria based on its scientific and economic impact (Mansfield et al. 2012). Like other *Xanthomonas* species, *Xam* has a particular group of proteins called transcription activator-like (TAL) effectors, which are able to bind directly to the host DNA and manipulate the transcriptional activity of target genes (Bogdanove et al. 2010). TAL effectors bind to DNA promoter regions of the plant called effector-binding element (EBE) in a particular base-specific fashion. TAL effector binding specificity depends on the presence of a central region consisting of 34–35 nearly identical residues repeated several times where the hypervariable 12 and 13 positions in each repeat are responsible for DNA recognition (Boch et al. 2009; Moscou and Bogdanove 2009). Once inside the plant nucleus, TAL effectors may activate susceptibility (S) genes to favor bacterial growth and dispersal and finally promote disease (Scholze and Boch 2011). Alternatively, naturally occurring EBEs are present in the promoter region of executor resistance (R) genes and function as molecular traps for TAL effector to induce host defense. The knowledge of the mechanism of action of TAL effectors opens new ways not only to improve CBB resistance but also to develop strategies to validate the function of candidate genes and even the possibility to direct cassava genome edition.

There are two main approaches to engineer plant resistance using TALs, first by modification or removal of natural EBE boxes from S gene promoters, and second by adding EBEs as molecular traps for the activation of executor R genes (Schornack et al. 2013). The first approach has been successfully used in rice for resistance against *Xanthomonas oryzae* strains, the causal agent of bacterial blight (BB). At least four different *X. oryzae* TAL effectors are known to target the disease S gene named SWEET14 to promote bacterial growth, suggesting a role of this gene as a major S gene for *X. oryzae* virulence (Yang et al. 2006; Antony et al. 2010). Using endogenous *X. oryzae* TAL effectors, TALENs (TAL effector nucleases) were developed to modify the EBE from SWEET14 promoter and render these plants resistant to BB (Li et al. 2012). In addition it was recently demonstrated that there is a natural variation in promoter targeting by TALs in rice germoplasm, some of which correspond to EBE and render the plant resistant to *X. oryzae* (Hutin et al. 2015). Nevertheless, in cassava no major S genes have been identified to contribute to *Xam* virulence. Current efforts using RNA-seq and the available EBE prediction tools may lead to the identification of putative S genes in cassava.

The second approach can be achieved by engineering a multiple TAL activator trap, consisting of multiple EBEs combined together in one promoter to induce the expression of a major R gene. This strategy may function as a broad-spectrum molecular trap since resistance will be induced by a diverse collection of strains or even different pathogens. In rice, the specificity of an executor R gene (*Xa*27) was broadened by the addition of multiple EBEs for *X. oryzae* to a single designed promoter (Hummel et al. 2012).

Once the sequence of any given TAL effector is known (the more prevalent, for example), it can lead to the artificial design of EBE boxes contained in promoters to activate executor R genes. In cassava, by using natural existing or artificial EBEs, it is possible to design synthetic boxes as traps to activate major R genes. Although in cassava no R or executor genes have been identified, the repertoire of typical immunity related genes predicted based on bioinformatics approaches have been reported and represent a “magic box” of sources of resistance genes (Lozano et al. 2015; Soto et al. 2015). Alternatively, the use of executor R genes isolated from other plants, such as *Xa*27, *Bs*3, or *Xa*10, can be considered a possibility.

In *Xam*, one TAL effector has been described in detail, named TALE1Xam (Castiblanco et al. 2013). This effector is present in several *Xam* strains, and it contributes to virulence in CFPB1851 (Castiblanco et al. 2013). Based on the code that determines nucleotide specificity, a strategy has been developed to induce expression of alternative executor R genes, such as auto-active nucleotide-binding leucine-rich repeats (NB-LRR) proteins to activate immune responses. In cassava, no *Avr*-R interaction have been described to date, thus the strategy of using promoter traps containing an EBE for TALE1*Xam* to activate putative R genes may be promising for cassava resistance to CBB.

### Nutritional improvement

Cassava is considered a poor source of micronutrients and proteins, especially white roots that are most commonly consumed fresh and/or for starch production. Several researchers have used genetic transformation to introduce genes to bio-fortify cassava, that is, to increase the content of macro- and micro-nutrients in roots. Expressing genes like CRTB, the bacterial version of plants’ phytoene synthases (PSYs), in white cassava roots of cv. 60444 increased total carotenoid content (TCC) up to 30 times (22–31 μg/g DW). The levels of pro-vitamin A, or β-carotene (BC) also went up to almost 7 μg/g DW, making former white roots appear orange (Welsch et al. 2010; Failla et al. 2012). Although the increase in TCC was outstanding, none of the transgenic lines of cv. 60444 could have outperformed conventionally bred lines with 70 μg/g DW TCC (Morillo et al. 2012), simply because the formers were transgenic and were also in a cultivar of limited use in Africa where biofortification efforts through biotechnology have been focused (Sayre et al. 2011). However, transgenic cassava carrying exogenous genes of the carotenoid pathway demonstrated that there were genetic bottlenecks in white roots that prevented them from accumulating BC. One such constraint was the absence of an efficient Phytoene Synthase enzyme able to synthesize phytoene, the first carotenoid of the pathway. That bottleneck was resolved with the overexpression of CRTB alone (Welsch et al. 2010), together with two more bacterial genes for phytoene desaturase (CRTI) and lycopene β-cyclase (CRTY; Bonilla 2010) or with the upstream gene deoxy-xylulose 5-phosphate synthase (DXS; Failla et al. 2012). The CRTB gene inserted and expressed in the white-rooted cv. 60444 was later moved through crossing with a yellow-rooted breeding line (GM905-21; Chavarriaga 2013) to obtain progeny overexpressing CRTB with TCC slightly higher than the yellow-rooted parent, i.e., 11 vs 8 μg/g DW, respectively, again, lower than that of the conventionally bred lines reported by Morillo et al. (2012). Nevertheless, altogether these researchers uncovered the presence of at least one more critical locus, different than PSY, to produce and/or accumulate carotenoids in the roots of cassava. Very recently SNP-based mapping analysis indicated that there are at least two sites in the cassava genome, in chromosomes two and seven, involved in BC accumulation (Ovalle et al. 2016). It is unknown if these loci are related to carotenoid synthesis, catabolism, or storage. This constitutes an excellent opportunity to use genome-editing tools to find out the true role of this and other genes of the carotenoid pathway in cassava.

When compared with *Brassica oleracea,* cassava roots are very deficient in iron, i.e., broccoli may contain up to 1089 mg/kg of iron while cassava’s iron content ranges from 4 to 49 mg/kg in roots (Mazilla-Dixon et al. 2015). The required daily intake (RDA) of iron in humans ranges from 8 to 18 mg of iron/d (White and Broadley 2005). If a person were to satisfy their RDA eating cassava, that person will require between 163 and 367 g DW of high-iron (49 mg/kg) cassava roots. If one assumes that the average dry matter content of cassava is around 30%, then, in terms of fresh root consumption, that same person may have to triple (489–1101 g) the quantity of cassava consumed to satisfy iron RDA. On the other hand, if the cassava variety consumed is in the lower range (4 mg/kg) of iron content, the amount of fresh roots required to satisfy the RDA becomes inedible (12–13.5 kg FW). This may be one of the reasons why researchers have developed transgenic cassavas with increased iron content in roots, i.e., Ihemere et al. (2012) and Narayanan et al. (2015; 2016), with the latter authors showing that levels of iron can be increased ten times in the cultivar TME204 when grown in the field, an interesting achievement considering that TME204 is a favorite of African farmers.

Vitamin B6 has also been enhanced by 4–48-fold in cassava leaves and 2–6-fold in roots of plants grown in fields by the overexpression of two *Arabidopsis* genes: *At*TDX1.1, a synthase, and *At*TDX2, a glutaminase for the biosynthesis of vitamin B6 in plants (Li et al. 2015). According to the authors, these plants can provide the RDA of 1.3 mg/d for an adult with as low as 51 g of boiled leaves or ten times more of boiled roots. For both, biofortified cassava plants with iron or vitamin B6, the necessary condition for their deployment and use in Africa is having resistance to CMV and CBSV. These two micronutrients are required in minute quantities and excessive consumption could cause toxicity (Fraga 2005; Hellmann and Mooney 2010) at least in model animal cell systems. It is therefore advisable to grow them under supervision, in specialized gardens, and perhaps under the protection of anti-whitefly mesh to prevent infection with viruses. Of course, an *in vitro* supply of clean material should be available to replenish plants whenever necessary.

### Propagation by tissue culture

Cassava is a highly heterozygous crop and many cultivars do not flower or do not produce enough, viable seeds. A system for vegetative propagation by stakes (stems or propagules) has therefore been developed to maintain traits of interest. This system presents advantages for the farmer since they can exchange stakes freely, but it comes also with disadvantages such as a low rate of propagation (7–10 new stakes/mature plant/cycle) with long waiting periods to get enough planting material (8–14 mo), delayed diffusion of new improved cultivars, and virus- and phytoplasma-associated disease dissemination. All of the above risks make the free exchange of germplasm between production areas unsafe. Most of these difficulties can be solved by using tissue culture technology. Small pieces of tissue can be used to increase the rate of propagation, under controlled aseptic conditions, without interference of climate, within short time frames and minimal space, thus enabling the establishment of seed producing systems for distributing disease-free planting material. A major difference with respect to conventional propagation by stakes is that *in vitro* technology reduces or abolishes spreading diseases causal agents, though the sanitary quality of planting material must always be verified before starting an *in vitro* multiplication program. Table 2 lists some of the technologies used for propagating cassava.

**Table 2.** Technologies for cassava propagation

Primary source of plants for propagation	Propagation system recommended^z^	
	Conventional	*In vitro*
Plants in the field	Stakes from mature stems, 2-node cuttings, and 1-bud-1-leaf cuttings	Rosettes
Botanical seeds^y^	Stakes	*In vitro* germination and/or embryo rescue for breeding
Meristems subject to thermo- or cryo-therapy to eliminate virus	n.a.	Grow on solid media low cost^x^ Bioreactors (i.e., RITA®)^w^
Somatic embryos	n.a.	Naked or encapsulated embryos (synthetic seed)
First generation of *in vitro* plants in field	Young plants (4 to 6 mo old)^v^; 2-node cuttings^v^; tunnels^v^	n.a.

^z^Any system of propagation must start from disease-free certified plants

^y^This is not a conventional multiplication system, although it may be used in the absence of basic planting material to initiate plantations. Plants with the best characteristics can be selected and propagated to increase numbers (Rajendran et al. 2000)

^x^Propagation of cassava can also be done using low cost, locally available, farmer-reachable inputs (Escobar et al. 2006; 2013a)

^w^Escobar et al. (2001a)

^v^Seed systems need planting material derived from tissue culture to scale-up cassava propagation by either one of these three methods (Escobar et al. 2012)

Conventional propagation can start from stakes cut from certified stems without going through *in vitro*. The process can be accelerated using simple modifications such as two-node and/or one-bud-one-leaf cuttings method (Patena and Barba 1971; Cock et al. 1982). In spite of the operational advantage and reduced production costs of conventional propagation, continuous water and power supply, and large areas for rooting stakes may be necessary depending upon the methodology implemented. On the other hand, *in vitro* propagation can be carried out in two ways: (1) either by growing or multiplying existing meristems or by (2) using *de novo* regeneration of plants via organogenesis and/or somatic embryogenesis. Roca (1979) developed a multiplication system for cassava known as rosettes, which involves meristems producing multiple shoots that look like cauliflower heads *in vitro*. Rosettes are actually intermediates between conventional and *in vitro* propagation since they start with large apical structures, 4 to 5 mm long, containing a meristematic dome and four- to six-leaf primordia, extracted from sprouting cuttings in the field and cultivated on medium supplemented with 6-benzyl amino purine (BAP). The role of BAP is to break up apical dominance for inducing multiple adventitious buds (Table 3). By adding gibberellins (GA3), the pre-induced buds elongate into numerous *in vitro* plantlets. Rosettes can produce up to eight times more plants than conventionally, *in vitro* propagated stems on solid medium, an attractive number to scale up cassava multiplication, provided that the phytosanitary status of the staring material is guaranteed. A drawback is the cultivar-dependency of the technology.

**Table 3.** Plant growth regulator composition of media for *in vitro* propagation, rooting, and conservation of cassava at CIAT

Medium component^z^ (μM)	4E	17N	8S	Rosette
GA_3_	0.1443	0.0288	0.2886	
6-BAP	0.1775		0.0887	2.2193
NAA	0.1074	0.0537	0.0537	0.0537

^z^The basic solution for all media, except 17N, contains the complete MS basal salt mixture (Murashige and Skoog 1962), m-inositol 554.93 mM, thiamine-HCl 2.96 mM, and sucrose 58.44 mM. Medium 17N contains instead 1/3 of MS salts plus 25 mg/l Plantex® (fertilizer N/P/K 10:52:10), with the other components kept constant.

Theoretically, routinely under *in vitro* conditions the number of plants expected (*Z*) can be calculated as *Z* = *XY* ^*n*^ − *L*, where *X* is the number of initial plantlets, *Y* is the rate of propagation observed (for solid-medium systems, cassava normally has a rate of 1:3–4), *n* is the number of cycles or generations carried out (8 cycles of 45 d, or 12 cycles of 30 d each/yr), and *L* represents losses due to contamination, no responding explants and no true-type plants (Escobar et al. 2012). Thus, under optimum conditions, one can expect between 6.5 × 10^3^ (*n* = 8) and 1.6 × 10^7^ plantlets/yr (*n* = 12) with a propagation rate (*Y*) of 1:3 to 1:4, respectively. The assumption is that there is no loss of plants due to contamination or other factors, which is never the case. Such numbers may be achieved following good sterility practices with well-trained manpower.

The multiplication of single nodes containing one apical or axillary bud each is the simplest way of propagating cassava *in vitro*. Cuttings are explanted on basal MS medium (Murashige and Skoog 1962), at 28 ± 2°C, and a 12 photoperiod with 18.5 μmol m^−2^ s^−1^ of light. The bud growth rate depends mainly on the balance between plant grow regulators (PGRs), temperature, and light. Two media are the most commonly used for this purpose: 4E, for apex or bud growth, and 17N for rooting (Roca 1984). During the first phase of an *in vitro* propagation cycle, the growth of buds is stimulated on 4E medium while, in the second phase, rooting of well-developed shoots occurs on 17N medium. The latter favors root development while slowing shoot growth (Escobar 1991). The interval between these two propagation cycles ranges between 30 to 45 d. CIAT has developed and used the single-node system to multiply the entire cassava world collection, 6467 clones from 28 countries (CIAT 2016). The collection is kept under trust and is considered the most important worldwide in terms of number of conserved accessions, the genetic diversity and geographic area coverage.

During the late 1990s, Konan et al. (1997) established an *in vitro* cassava propagation system that pre-induced a multi-meristematic structure, i.e., shoot tips, using high concentrations of BAP (22–44 μM) and the surfactant Pluronic® F-68 (2%, *w/v*) on solid media. The authors obtained propagation rates five times higher than those obtained with the internode system. Similarly, De Oliveira et al. (2000) established a mass propagation system using technology developed by EMBRAPA for six Brazilian varieties. The propagation rate after 30 d was 1:2.9, regardless of variety and number of subcultures. Other than starting with disease-free plants, these systems do not seem to offer an advantage in terms of numbers to scale up cassava multiplication for commercial purposes. Fortunately, Alvard et al. (1993) described a large-scale, temporary immersion system for plant propagation known as RITA®, which proved successful for somatic embryogenesis and organogenesis of species like banana, coffee, rubber, pineapple and sugar cane. With support from the Colombian Corporation for the Participatory and Sustainable Development of Small Farmers (PBA Corporation), CIAT adjusted, validated, and implemented RITA® for cassava, using 20 commercial varieties from diverse production areas in Colombia. Propagation rates increased to 1:6–23 after 45 d, depending on the variety (Escobar et al. 2001a; b), making RITA® the most efficient system for mass production of cassava plants *in vitro*. Even if hyper hydrated plantlets were produced, which is common, they could recover normal appearance by alternating cycles of multiplication on solid/liquid media.

### Producing pathogen-free stocks via tissue culture

The safe movement of cassava germplasm requires certified virus-free plants. Quarantine protocols apply for detecting CMD, CBSVD, CFSD, cassava common mosaic virus (CCMV), cassava virus X (CsVX), cassava American latent virus (CALV), and cassava Colombian symptomless virus (CCSpV) (Frison and Feliu 1991) in shipments of cassava plants. Hence, strategies must be implemented for cleaning plants using thermotherapy. This is usually carried out by the application of a heat regime to whole plants for 3–4 wk, at 40°C during the day and 35°C at night, 80% relative humidity and 12-h day length (Roca and Jayasinghe 1982). Meristems are then extracted for proliferation and to establish *in vitro* banks. Finally, the absence of virus must be certified by indicator plants and molecular tools like PCR-based detection and/or ELISA kits when available. An alternative method for freeing plants of virus is through somatic embryogenesis, which has been proved in cassava for ACMV, EACMV, and CMV (Damba et al. 2013; Nkaa et al. 2013).

The drawback of tissue culture-mediated, virus-free certification of planting material may reside in the genotype dependency and induced epigenetic variation intrinsic to *in vitro* culture. Simple propagation by nodes *in vitro* alters the methylation pattern of plants when compared with stakes grown in the field (Kitimu et al. 2015). This variation may be undesirable or, on the contrary, it may be another excellent source of variability to exploit for epigenetic breeding in cassava, as it has been the case for tomato (Yang et al. 2015). Although differential methylation patterns near genes do not necessarily mean changes in gene expression with subsequent changes in phenotypes, the implications for cassava are unknown. There is an urgent need to understand and either exploit or prevent differential methylation.

Although cryotherapy involves *in vitro* culture and therefore possible epigenetic effects, it is a new method to eradicate pathogens in cassava using shoot tips. Aranzales (2013) used single tips from *in vitro* plants to apply three 15-d cycles of thermotherapy (40°C day/35°C night) before applying a droplet vitrification method (Escobar et al. 2014) to eliminate CCMV and a Reovirus associated with CFSD.

### Somatic embryos and synthetic seeds for multiplication and disease-free planting material

Synthetic seeds are defined as somatic embryos enclosed in artificial nutrient media that can be stored and germinated to produce whole plants, thus mimicking the function of sexual seeds to propagate plants (reviewed by Reddy et al. 2012). Virus-infected cassava plants reduce yield substantially (Legg et al. 2014), but cassava plants can be freed of viruses after passing through a cycle of somatic embryogenesis (Nkaa et al. 2013), which encourages the use synthetic seeds and/or somatic embryos for cleaning and massive propagation. Further, compared with conventional systems of clonal reproduction, synthetic seeds and somatic embryos have the advantage of always being free of pathogens and virus because they must come from certified *in vitro* plants. In addition, both can be produced in large quantities with minimum space and manpower requirements and free of rough climate variations. The space and amount of nutrient media required to grow 1 g of embryogenic cells (potentially 1000 somatic embryos) takes only five Petri plates (9 mm diameter each) and 63 d. This scale of somatic embryo production uses neither bioreactors nor liquid medium for upscaled propagation leaving more room for improvement (Martínez et al. 2014). Besides, it also assumes that variables like embryo morphology, genotype, encapsulation, and cold storage, among others, reduce the efficiency of somatic embryo germination by 50%, so the estimated final embryo-to-plant conversion rate is actually 2:1 (two embryos give rise to one plant). There are reports claiming that from 1 mg of embryogenic cells, 1.5 to 8 plants can be obtained *in vitro* (not established in the greenhouse; Raemakers et al. 2001). A germination range between 40% and 80% indicates that the technology can be substantially improved. In reality, propagating clonal seeds from somatic embryos is not as straight-forward as it may seem and requires optimization. The production of cassava plants from milligrams of embryogenic cells could become much more efficient if, for example, liquid (RITA®) instead of solid media were used, facilitating the synchronization of somatic embryo development. The automation of somatic embryo production using liquid systems in bioreactors requires standard environmental conditions like pH, temperature, light, O_2_ supply, CO_2_ exchange, media composition (i.e., growth regulators), and antibiotic depletion, among other variables (Ducos et al. 2007). Coffee is an excellent example: bioreactors facilitate the massive production of 2–4 × 10^5^ somatic embryos in 2–5 l bioreactors for direct sowing in the greenhouse, without encapsulation. This approach is worthwhile trying for cassava (Ducos et al. 2007; 2011; Etienne et al. 2013).

**Figure 2.**
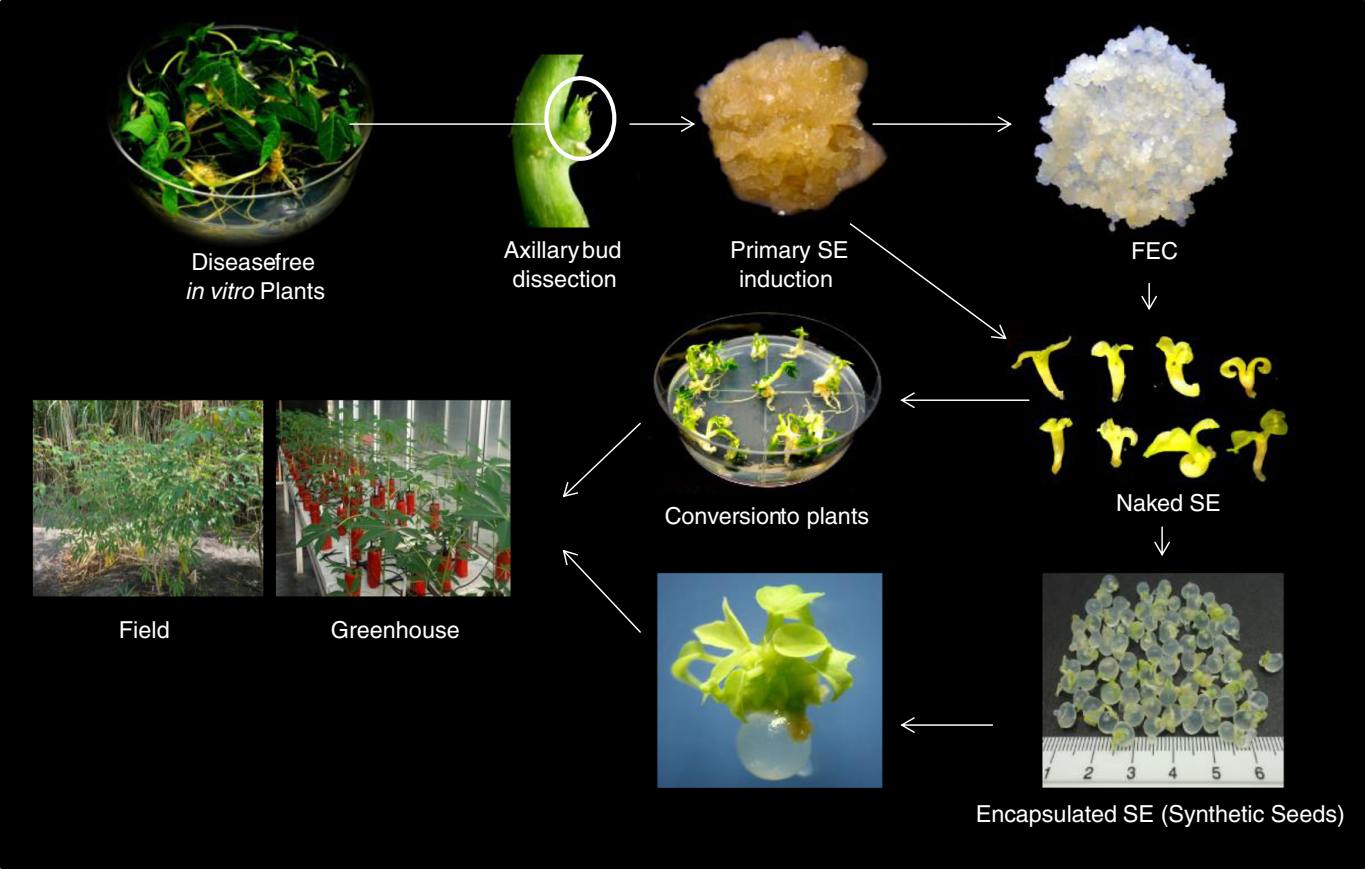
Flowchart for the production of cassava plants from naked or encapsulated somatic embryos (synthetic seeds). The initial source of explants must be certified, disease-free, *in vitro* plants (*upper left corner*), from which axillary buds are dissected to induce primary somatic embryos (*primary SE*) or friable embryogenic callus (*FEC*). SEs can then be used with an artificial coat (*encapsulated SE*; *scale* in cm) or without it (*naked SE*) for producing plants *in vitro*. Encapsulated SEs are equivalent to synthetic seeds which, as described in the text, may be used for short-term storage of cassava germplasm. It is unknown if synthetic seeds tolerate below-freezing temperatures, which would be ideal for the long-term storage of germplasm.

*In vitro* protocols for the encapsulation and cryopreservation of nodal cuttings (axillary buds) and shoot tips (apical buds) have been reported in cassava (Escobar et al. 1997; 2001b, 2013b, Danso and Ford-Lloyd 2003; Charoensub et al. 2004) for germplasm preservation and exchange. However, multiplication rates of cassava plants *in vitro* do not compete with those of somatic embryos; embryogenic cells in bioreactors double their mass every 15 d (Ducos et al. 2007). Furthermore, the isolation of axillary buds or shoot tips requires manual, individual handling, which increases operations and cost and hinders automation for encapsulation and storage. On the other hand, use of synthetic seeds and primary somatic embryos definitively reduce steps in the production of propagules for cassava multiplication (Fig. 2). The prospect of automated mass production as in coffee, with up to 4 × 10^5^ somatic embryos l^−1^ (Ducos et al. 2007), is very appealing for cassava propagation. Several cassava cultivars produce thousands of somatic embryos or FEC on a single semisolid medium using the synthetic auxins 2,4-D or Picloram (Taylor et al. 2001; 2004; Liu et al. 2011). There is a high potential to use SE as a source of propagules for scaling-up cassava multiplication or for cryopreservation for the long-term storage of cassava germplasm.

From CIAT’s own experience, one somatic embryo-derived cassava plant can be established from 1 mg of totipotent cells every 63 d (Martínez et al. 2014). When embryos of cultivar SM1219-9 were encapsulated, germination was found to be genotype and stage dependent, with best germination reaching up to 90% for early-cotyledon embryo stages. For other cultivars like Tai16 and 60444, germinations were 53% and 19%, respectively. Cold storage of synthetic seeds of cultivar SM1219-9 (5°C for 20 d) reduced germination rates to a maximum of 33%, although plants were still recovered. Antibiotics did not have detrimental effects on germination of synthetic seeds with no cold treatments. Encapsulated shoot tips germinated at rates closer to 100%, again, without cold storage (Martínez et al. 2014). The extraordinary multiplication rates of somatic embryos, allowing automated mass production of plants via somatic embryogenesis and synthetic seeds, constitutes a powerful alternative to other vegetative propagation techniques for producing pathogen-free, certified cassava plants.

**Figure 3.**
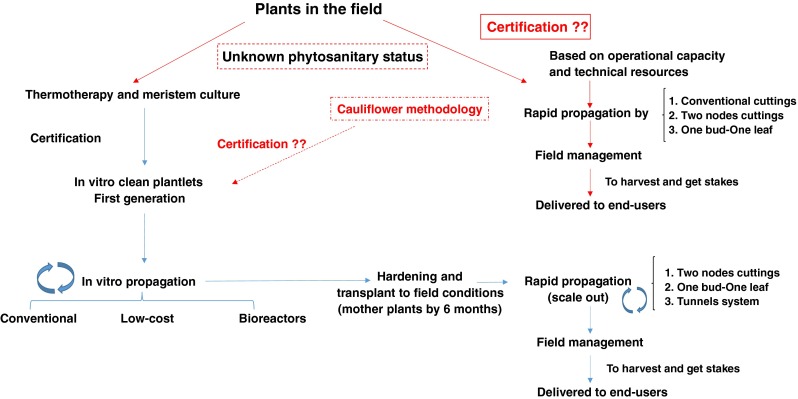
Flowchart for decision making on propagation methods for cassava planting material production. The success of diagram procedures connected by *red lines* depends solely on the initial material certification as disease free. The lack of such certification results in lack of confidence in the system and may result in the distribution of unhealthy planting material in farmers’ fields. One possibility to ensure clean starting material is that gene banks provide certified plants *in vitro*. Any cassava seed system (*blue arrows*) must integrate *in-vitro* platforms with macro-propagation schemes to offer high-quality abundant planting material continuously to the end-users.

In short, fast production of enough and high-quality planting material for cassava is technically possible using a diverse array of biotechnological *in vitro* tools, some of which were explained above and are summarized in Fig. 3. The prerequisites for the successful implementation of any seed distribution system, conventional or biotech-based for cassava are the same: it must start from pathogen-free, certified planting material; the cost per plant should be affordable by users, be them small-scale farmers or seed producers; it may be decentralized and must be supported by official agricultural agencies since private companies may not be interested in developing seed systems for cassava. Finally, it must provide new, higher-yielding varieties regularly, adapted to changing environmental conditions and consumer demands.

## Perspective and Concluding Remarks

The landscape of biotechnology applied to improve cassava has been encouraging given the number of traits and cultivars that entered the pipeline of biotech-based breeding in the last 20 yr. It is remarkable that laboratories in developing countries have been able to adapt and adopt biotechnology as a tool for breeding. However, although we have passed the stage of proof of concept for traits like virus resistance, we still expect GM varieties released in Africa to combat CMD and CBSD. While the problem of loss of natural resistance to CMD gets solved, we must keep thinking on *in vitro* propagation as the more reliable source of virus-free plants. There is an urgent need to generate more efficient systems for multiplication and hardening of plants, in each country, probably decentralized but with the support of governments, extensionists, and tissue culture specialists, similar to what exists today for the cocoa and timber industry.

There have been few examples of development of rapid propagation, delivery and storage of crops using synthetic seed technology since the late 1970s. Significant progress in Douglas-fir and loblolly pine has been achieved due to the upscaling of SE in bioreactors and the design of new encapsulation protocols. For cassava, synthetic seeds may be viable if protocols minimize the dependency on genotypes to produce abundant SE/FEC in varieties of economic interest, followed by scaling up SE/FEC using bioreactors and finally ensure genetic and phenotypic stability of plants generated from these seeds. It is noteworthy that most low-scale farmers demand free, disease-free planting material. This translates into having to produce synthetic seeds at low cost, which is a potential limiting factor given that industries, which have set up seed systems for other clonally propagated crops, may not be interested in developing them for cassava multiplication. Implementing the technology described in this review will therefore be costly. National agricultural research programs (NARPs) rely heavily on clonal propagation of cassava by stakes often under uncertified phytosanitary conditions. The phytosanitary conditions are difficult to maintain after cycles of propagation in the field. Is this trend changing soon? Can NARPs and the seed industry envision safer, faster, and cheaper propagation systems for cassava? The technology exists, but the willingness may not.

Biotechnological efforts continue to improve traits in cassava, some mentioned in this review such as biofortification of roots and leaves, SLCMV resistance for KU50, waxy cassava (reviewed by Liu et al. 2011), haploid induction, and glyphosate- or PPT-tolerant cassava (Table 1; Chauhan et al. 2016), among others. All these transgenic plants may be seen as final products for countries where African viruses are not yet a constraint. They must be seen also as a source of new genes, new traits for breeding programs worldwide. Biotechnology is a tool to rapidly add genetic variability and/or to discover the genes behind the features. As said before, several years and funds have been spent searching for a biotechnology-derived product in cassava. There are several transgenic prototypes that, with more investment and less regulation and less opposition, could be pioneer germplasm. Unfortunately the anti-GM trend has influenced and will largely determine whether we should keep waiting. Is genome editing a valid alternative to transgenesis to develop the long-awaited, biotechnology-derived, non-GM products in cassava? The tendency in the USA may indicate that genome-edited crops (reviewed by Hsu et al. 2014; Osakabe and Osakabe 2014; Bortesi and Fischer 2015) may be the way to go. They are or may be released soon and are not considered genetically modified, therefore free of the regulation imposed for transgenics. For example, herbicide-tolerant canola (CIBUS 2016), mushrooms with reduced oxidation (Waltz 2016), and waxy corn (DuPont-Pioneer 2016), have edited traits very relevant for cassava breeding; even for adding resistance to DNA and RNA viruses (i.e., Pricea et al. 2015), and the list may be endless. Therefore, a priority will be the standardization of genome editing methods applicable to several cassava varieties that guarantee the maintenance of the genotype and produce non-transgenic varieties. The successful implementation of this technology will depend on public acceptance, for which the challenge is also an anticipated, truthful, accurate and fast communication with the general public, and with non-scientists and decision makers in governments.

Last but not least, to the question if climate change will affect global cassava production, the answer is uncertain. Obviously, it will depend on the region and the period to which we refer. In their work on modeling climate change and its impact on cassava in Africa, Jarvis et al. (2012) predict that, compared with beans, potatoes, bananas, and sorghum, cassava will have a much lower percentage of negative impact change (−3.7%) in climate suitability or the ability of the crop to produce under new climatic conditions. By way of comparison and according to this same study, the estimated negative impact on beans and potatoes in Africa would be −16% and −14.7%, respectively. The reason why climate change would not have such a severe impact on cassava production in Africa is that, in countries where it is mostly grown (East, West and Central Africa), the maximum temperature rise for 2030 is forecasted to be below 1.5°C, a tolerable change for a crop adapted to drought and high tropical temperatures. However, climate change will not be uniform across the globe and will possibly open new niches for biotic and abiotic stresses decimating current cassava production. For cassava farmers in South China, South Africa, Southern Brazil, Paraguay, and Northern Argentina, tolerance to low temperatures and even frost is crucial, so conventional and biotechnology-mediated breeding should keep focused on improving this trait. Climate change also opens new niches for whiteflies, for example, by heating areas where they could not inhabit before. These CMD-transmitting insects are highly adaptable to changes in temperature by altering the expression pattern of heat shock proteins, which allows them, among other effects, to increase fitness after heat shocks (Díaz et al. 2015). Thus, these new “windows” opened by climate change that would affect cassava production for the next 30 yr, may be paradoxically perceived as windows of opportunity for improving cassava through biotechnology, hand to hand with conventional breeding, an initiative that started over 30 yr ago and remains current.
